# Plant Genebanks: Present Situation and Proposals for Their Improvement. the Case of the Spanish Network

**DOI:** 10.3389/fpls.2018.01794

**Published:** 2018-12-04

**Authors:** María José Díez, Lucía De la Rosa, Isaura Martín, Luís Guasch, María Elena Cartea, Cristina Mallor, Joan Casals, Joan Simó, Ana Rivera, German Anastasio, Jaime Prohens, Salvador Soler, José Blanca, José Vicente Valcárcel, Francesc Casañas

**Affiliations:** ^1^Mixed Unity for the Valorization and Breeding of Horticultural Landraces, Institute for the Conservation and Improvement of Valencian Agrodiversity, Universitat Politècnica de València, Valencia, Spain; ^2^Spanish Plant Genetic Resources National Center, National Institute for Agricultural and Food Research and Technology, Alcalá de Henares, Spain; ^3^Group of Genetics, Breeding and Biochemistry of Brassicas, Misión Biológica de Galicia, Spanish Council for Scientific Research, Pontevedra, Spain; ^4^Agrifood Research and Technology Centre of Aragón, Agrifood Institute of Aragón, University of Zaragoza, Zaragoza, Spain; ^5^Mixed Unity for the Valorization and Breeding of Horticultural Landraces, Miquel Agustí Foundation. Department of Agrifood Engineering and Biotechnology, BarcelonaTech, Castelldefels, Spain; ^6^Department of Plant Breeding, FITO Seeds, Barcelona, Spain; ^7^Institute for the Conservation and Improvement of Valencian Agrodiversity, Universitat Politècnica de València, Valencia, Spain

**Keywords:** genetic variability, phenotypic variation, *ex situ* conservation, landraces, crop wild relatives, seedbanks, gene conservation, plant genetic resources

## Abstract

Genebanks were created by the middle of the twentieth century to preserve cultivated biodiversity when landraces began to be substituted by modern varieties. This move was generally accepted as a necessary step to safeguard the future. After about 75 years of collecting and maintaining genetic resources, the increasing ability of biotechnology to create new variability brings the roles of genebanks in the present and near future into question. As a continuation of several workshops that started in 2014, staff of some representative genebanks have met to discuss how the Spanish Plant Genetic Resources Network can be improved, identifying the following major shortcomings: lack of efficient coordination in the distribution of species among genebanks; too many genebanks; existence of detected and undetected duplicates; insufficient rate of regeneration; insufficient phenotyping, genotyping, and epiphenotyping; unsatisfactory rate of use by end users; and, insufficient funding. As a considerable increase in public funding is unlikely, we propose some strategies to increase the efficiency of the system. The most urgent tasks are to strengthen the rationalization of the network by establishing a clear hierarchy and functions, to improve the information in the base collection by deep characterization including not only phenotypes but also uses and utilities, to progressively replace the active collections with focused core collections constructed to meet users' needs, to optimize regeneration protocols, to limit new collecting expeditions of Spanish crop wild relatives to those growing in threatened habitats, and to develop user-friendly platforms to access germplasm documentation, including a unified system of descriptors and classification categories. Current advances in biotechnology, and especially those in gene editing will have without doubt an impact on the role of genebanks. However, the high number of genes and gene combinations created by evolution they hold cannot be produced by these techniques at present. So, these reservoirs of variability will continue to be indispensable for the near-medium future while the function of all the genes is unveiled. In turn, biotechnologies and gene editing will allow us to take advantage of the information held in genebanks in a more efficient and fast way, contributing to a better rationalization and functioning.

## Introduction

The *ex situ* conservation of plant genetic resources started by the mid-twentieth century as a reaction to the rapid loss of agricultural biodiversity, mainly due to the replacement of landraces by improved varieties (Gepts, 2006; Van de Wouw et al., 2009; Khoury et al., 2014). This replacement was made possible by enormous energy inputs into agrarian systems in the form of machinery, fertilizers, pesticides, herbicides, irrigation, protected cultivation, etc., which make environmental conditions more uniform, thereby allowing a limited number of improved varieties to be grown everywhere, replacing landraces adapted to microenvironments, local cultivation methods, and cultural elements of use. It has been estimated that 70% of currently cultivated crops are of foreign origin, while the traditional crops indigenous to each area are disappearing (Khoury et al., 2016).

When genebanks were created, they were intended to preserve genetic material (fundamentally gene combinations) with the aim that they might be used in the future (Fowler and Hodgkin, 2004), either directly or as material in breeding programs (Tanksley and McCouch, 1997) to face potential changes in environmental conditions or societal needs, even before discussions about climate change started. After decades of experience with genebanks, the advantages and disadvantages of this strategy with respect to conservation *in situ* have been discussed extensively [(Gómez et al., 2005; Gepts, 2006; Veteläinen, 2009; Negri and Tiranti, 2010)]. To date, it seems that *ex situ* conservation has had more success than *in situ* conservation, probably because of its lower cost (about 100 times less than *in situ* conservation; De-Zhu and Pritchard, 2009) and greater ease for users to access the material. Since the first plant genebanks were established, biological technologies have evolved immensely, so breeders now have more tools available to generate variability and also new data sources. Thus, it is time to evaluate to what extent genebanks' main functions (collection, documentation, regeneration, distribution, and conservation) should be reconsidered.

The Iberian Peninsula's diverse climates (Mediterranean, Atlantic, Continental, and Alpine) and soil types, together with its location and history, have made it an important center of agricultural diversity. Its proximity to the area of domestication of the Fertile Crescent, and colonization by the Muslims facilitated the arrival of plants from Asian and African domestication zones (Hancock, 2004). Later, its participation in the colonization of America also made it an expansion zone for many American cultivated plants and a secondary center of diversity for some of them, such as the tomato *Solanum lycopersicum* L.; (Cebolla-Cornejo et al., 2013) or common bean (*Phaseolus vulgaris* L.; Santalla et al., 2002). These circumstances have generated a wealth of landraces, which are largely preserved in genebanks.

This paper aims to review the system of Spanish genebanks to: (i) examine their activities, (ii) review the present and expected demands of society on genebanks, and (iii) evaluate to what extent genebanks' activities and their organization should be modified to optimize their present work and to adapt them to meet new needs. An analysis of the situation in our country should help firstly in rearranging national system but can be also considered within an international perspective.

## Genebanks in Spain

The first Spanish genebanks, derived from breeders' seed banks, were linked to research institutions, mainly the regional centers of the National Institute for Agricultural and Food Research and Technology (INIA) devoted to regional crops. Rather than unique to our country, this situation is the general rule around the world, as many of the most important genebanks started in this way (Engels and Thormann, 2007). In the mid-1980s, political decisions resulted in the partial transfer of the INIA's tasks to regional governments, although the Program for the Conservation and Utilization of Plant Genetic Resources National Network (PCURF), established by Ministry of Agriculture in 1993, maintained a certain level of coordination. Later, new initiatives appeared, including regional banks and new public and private banks set up by breeders and conservation organizations.

**Figure 1:**
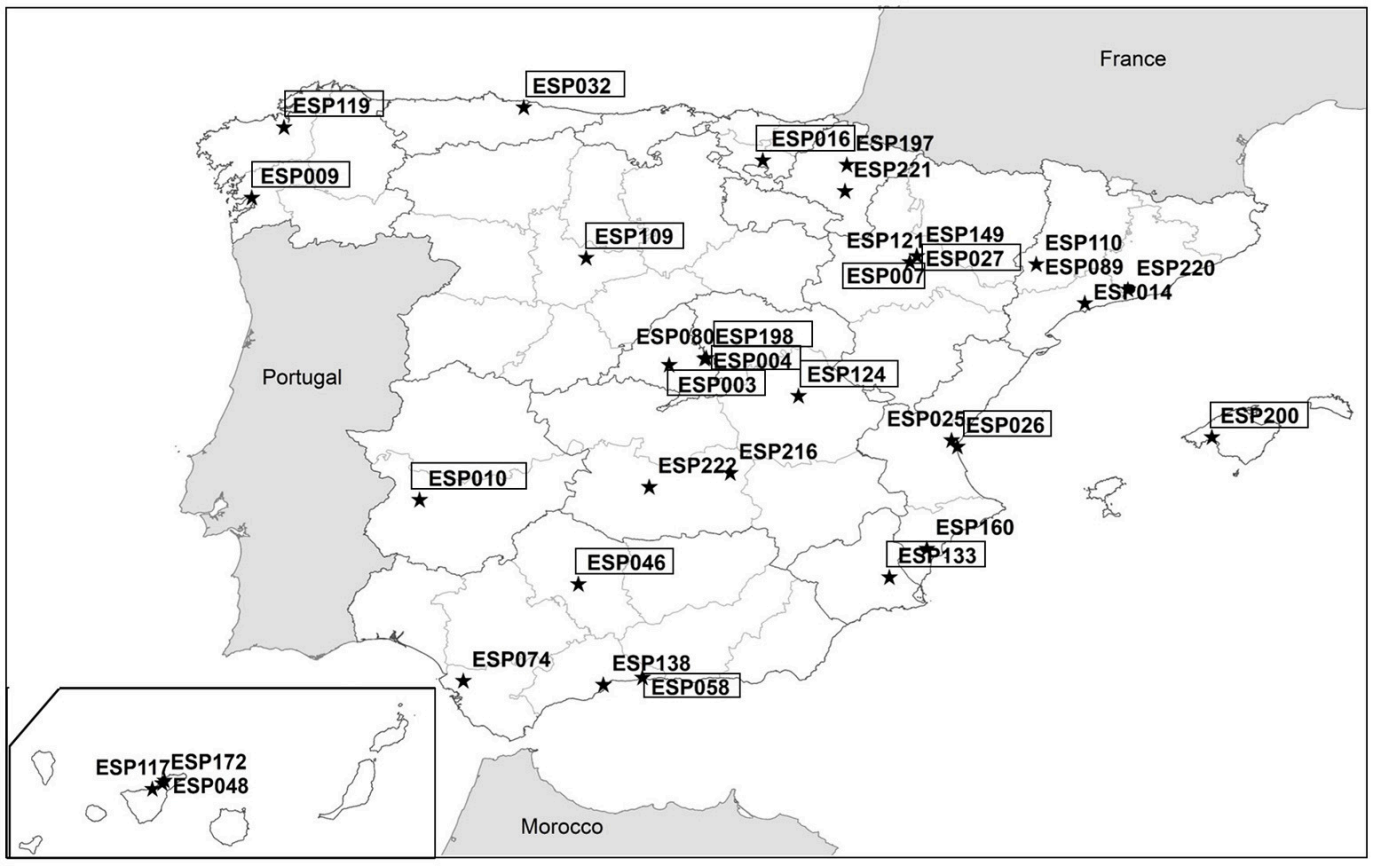
Location of the genebanks included in the Conservation and Utilization of Plant Genetic Resources Spanish Network. Institutions with framed codes store exclusively seeds or seeds and vegetative propagated crops.

The Spanish Plant Genetic Resources National Center (CRF), dependent on the INIA, is entrusted by law with the mission of preserving backup copies of the seeds from all Spanish institutions and managing the Collection Network (that includes also vegetative propagated material) and the National Inventory of Plant Genetic Resources (Figure 1 and Table 1; the readers are referred to Table 1 for detailed information about the acronyms cited in this section), in addition to its role as active collection of grain legumes, winter cereal and industrial crops. Then, other genebanks maintain active collections of national scope for specific crops or groups of crops, such as the COMAV and the CITA, which hold horticultural crops, or the CICYTEX, which hold forages/grassland and lupine crops (Figure 1 and Table 1). Additionally, there are banks that are clearly devoted to specific regions (e.g., IMIDRA in Madrid, SERIDA in Asturias, IMIDA in Murcia, CCBAT in the Canary Islands), others that maintain collections oriented toward breeding (MBG-CSIC and IHSM-UMA-CSIC) (Figure 1 and Table 1). Besides, in close connection with the network, there are still others that combine regional collections with breeding activities (Fundació Miquel Agustí in Catalonia, FMA). Finally, there are the community genebanks that belong to conservation organizations, which have a local scope and frequently do not follow the standard procedures for collecting, documenting, and conserving materials. On the other hand, seed companies have their own banks with materials they have obtained for or from different breeding programs, although it is difficult to know exactly what materials they have, since they are not freely available.

**Table 1:** Institutes in the Spanish Genebanks Network, full name, acronym, institute code, and kind of crops preserved in the banks.

**Instcode**	**Acronym**	**Seeds (S), vegetative propagated crops (V)**	**Name**
ESP003	UPM-BG	S	Genebank “César Gómez-Campo”
ESP004	INIA-CRF	S	National Center for Plant Genetic Resources
ESP007	CSIC - Aula Dei	S, V	Spanish Council for Scientific Research at Experimental Station “Aula Dei"
ESP009	CSIC-MBG	S, V	Spanish Council for Scientific Research, at the Misión Biológica of Galicia
ESP010	CICYTEX	S,V	Agriocultural Research Institute “La Orden-Valdesequera”
ESP014	IRTA-MB	V	Institute of Agrifood Research and Technology, Center “Mas Bové”
ESP016	NEIKER-Tecnalia	S,V	The Basque Institute for Agricultural Research and Development
ESP025	IVIA	V	Valencian Institute of Agricultural Research
ESP026	COMAV	S	Genebank of Institute for the Conservation and Improvement of Valencian Agrodiversity
ESP027	CITA-HOR	S	Vegetable Genebank of Aragón
ESP032	SERIDA	S,V	Regional Service for Agri-food Research and Development
ESP046	IFAPA-COR	S,V	Andalusian Institute of Agricultural and Fisheries Research and Training. Center “Alamada del Obispo”
ESP048	ICIA	V	Canarian Institute of Agricultural Research
ESP058	CSIC-La Mayora	S,V	Superior Council for Scientific Research at the Experimental Station “La Mayora”
ESP074	IFAPA-CAD	V	Andalusian Institute of Agricultural and Fisheries Research and Training. Center “Rancho de la Merced”
ESP080	IMIDRA-Vid	V	Institute of Research and Rural Development from Madrid
ESP089	UdL	V	School of Agricultural Engineering. University of Lleida
ESP109	ITACYL	S	Research Center of Zamadueñas
ESP110	CITA-FRU	V	Fruitculture Department of Aragon
ESP117	ICIA-La Orotava	S	La Orotava Acclimatization Gardens
ESP119	CIAM	S,V	Agricultural Research Center of Mabegondo
ESP121	GOB ARAGON	V	Rural Development and Sustainability Department of Aragon
ESP124	IRIAF-Albaladejito	S,V	Agricultural Research Center of Albaladejito
ESP133	IMIDA	S,V	Murcia Institute of Agri-Food Research and Development
ESP138	IFAPA-MAL	V	Andalusian Institute of Agricultural and Fisheries Research and Training. Center “Churriana”
ESP149	CITA-FOR	V	Forest Resources of Aragón
ESP160	UMH	V	Polytechnic High School. University Miguel Hernandez
ESP172	CCBAT	S,V	Centre for the Conservation of Agricultural Biodiversity in Tenerife
ESP197	UPN	V	School of Agricultural Engineering. Public University of Navarra
ESP198	IMIDRA-Variedades locales	S	Genebank of landraces of Madrid
ESP200	IRFAP	S,V	Institute of Agricultural and Fisheries Research and Training of Balearic Islands
ESP216	IRIAF-Vid	V	Institute of Vine and wine of Castilla La Mancha
ESP220	INCAVI	V	Catalonian Institute of Vineyard and Vine
ESP221	EVENA	V	Viticulture and Enology Station of Navarra
ESP222	IRIAF-Chaparrillo	V	Agricultural Center of “El Chaparrillo”

The reflections we present here are based on the data from a group of five genebanks dedicated to seed conservation: CRF, CITA, COMAV, MBG-CSIC, and FMA. The materials in these banks represent about 85% of the 62,470 accessions stored in the seed genebanks included in the National Network (without considering the base collection of the CRF). Here we describe their main characteristics.

The CRF genebank (http://wwwx.inia.es/coleccionescrf/) (Table 2), the most important bank in Spain, dates back to 1977 (Bueno and Alaman, 1982). It currently coordinates the country's plant genetic resources, conserves the base collection containing a backup copy of more than 43,000 seed accessions, and is the national documentation center. The CRF also maintains the active collection of legumes, cereals, and species for industrial use, which contains more than 22,000 accessions from 233 species. With a long experience in collecting missions (>35), it is currently the only institution that conducts collecting activities, especially targeting wild relatives of cultivated plants (CWR); it has the capacity to regenerate over 700 accessions per year in collaboration with other institutions. On average, between 2013 and 2017 it delivered 1,362 samples per year in response to 61 requests per year, most of which came from researchers and farmers.

**Table 2:** Relevant information on the genebanks surveyed.

**Genebank**	**Foundation year**	**Place**	**Crops**	**Number of accessions**	**Conservation conditions**	**Number of accessions regenerated per year**	**Online request form**	**Available information online**	**Number of accessions delivered per year (mean of 5 years)**
CRF	1977	Alcalá de Henares	All crops (seeds) ^*a*^	42,586 ^*a*^	−18°C^a^	657 with other institutions	Yes	Passport and partial characterization	1,375
			Leguminous and cereals ^*b*^	22,041 ^*b*^	−4°C^b^				
BGHZ	1981	Zaragoza	Cultivated and neglected vegetables crops	17,461	−18°C	190 with other institutions	Yes	Passport	600
COMAV	1981	Valencia	Cultivated vegetable crops and wild relatives	13,556	4°C	200 with other institutions	No	Passport and partial characterization	2,700
MBG-CSIC	1985	Pontevedra	Vegetable Galician *Brassica* crops	644	4°C	40–50	No	No	70
FMA	1992	Barcelona	Catalonian vegetable crops	1,774	4°C	100	No	No	50

a Base collection

b Active collection

CITA (https://sites.cita-aragon.es/BGHZ/) and COMAV (Table 2) are the two main active genebanks for horticultural crops. CITA began operations in 1981 and currently maintains a collection of more than 17,000 accessions of 245 species of horticultural crops, underutilized species, and CWR. With help from other institutions it regenerates about 190 accessions per year. Between 2013 and 2017, it distributed an average of 600 accessions per year, mostly to farmers and researchers. It collaborates with numerous associations of farmers, transferring materials and providing advice to the sector.

The genebank of the COMAV (https://www.comav.upv.es/index.php/databasesgermplasm/bancoger) (Table 2) began its collecting missions in 1981 and it currently has a collection of about 13,000 accessions of almost 200 different species. With the collaboration of IMIDA it regenerates some 200 accessions per year. Between 2013 and 2017, it distributed an average of 2,700 accessions per year, mainly to researchers, seed companies, and farmers. The COMAV participates in various European projects and maintains collaborations with numerous farmers and seed companies.

The *Brassica* genebank of MBG-CSIC (http://www.mbg.csic.es/es/) (Table 2) started its activities in 1985. In the present paper, we will focus only on the collection of Galician *Brassica* crops belonging to the species *Brassica oleracea* L., *B. rapa* L. and *B. napus* L., which houses 644 accessions. *B. oleracea* includes kales *(B. oleracea* var. *acephala*), cabbages (*B. oleracea* var. *capitata*), and Tronchuda cabbage (*B. oleracea* var*. costata*). *B. rapa* groups the turnips, turnip greens, and turnip tops; and *B. napus* appears only in the form known as “nabicol” or leaf rape. The difficulty of the controlled multiplication of these crops (allogamous with strong inbreeding effect) and their long growth cycles, limit the institution's regeneration capacity to about 40–50 accessions per year. It is an important collection of *Brassica* germplasm, adapted to the Atlantic conditions with high intra- and inter-variety variability (Cartea et al., 2003; Soengas et al., 2008). More than half of the requests come from farmers and associations, and the rest come from national and foreign research centers.

The newest genebank considered belongs to the FMA (http://fundaciomiquelagusti.com/) (Table 2). It started its activities in the late 1990s with efforts to recover the common bean (Casañas et al., 1997). The bank keeps 1,774 accessions of 25 different species. Its most important collections are of tomato (884 accessions) and Ganxet common bean (627 accessions). The collection consists almost exclusively of landraces collected in Catalonia (Casals et al., 2017). For most of the materials, data are available on agronomic and morphological traits. For some materials, sensory and/or chemical data related to organoleptic traits are also available. Initially, the collection was entirely dedicated to varietal recovery programs carried out in collaboration with farmers and cooperatives; however, the current collection is a representation of the genetic variability of the area, so it tends to function as a regional horticultural genebank.

The functioning of genebanks has come under discussion, with publications by Fu (2017) and Byrne et al. (2018), among others. In February 2018, representatives of the aforementioned banks met to discuss their main concerns and, by extension, those of the Network of Spanish genebanks. The aim was to examine to what extent the banks were fulfilling the challenges of meeting the changing needs of their scientific, technical, and social environments and to evaluate possible changes in their activities to assume new roles. We will focus only on genebanks holding seeds, as the problems associated with their management can be different from those of genebanks conserving vegetative propagated crops.

## System Weaknesses, Evaluation of Alternatives and Recommendations

**Figure 2:**
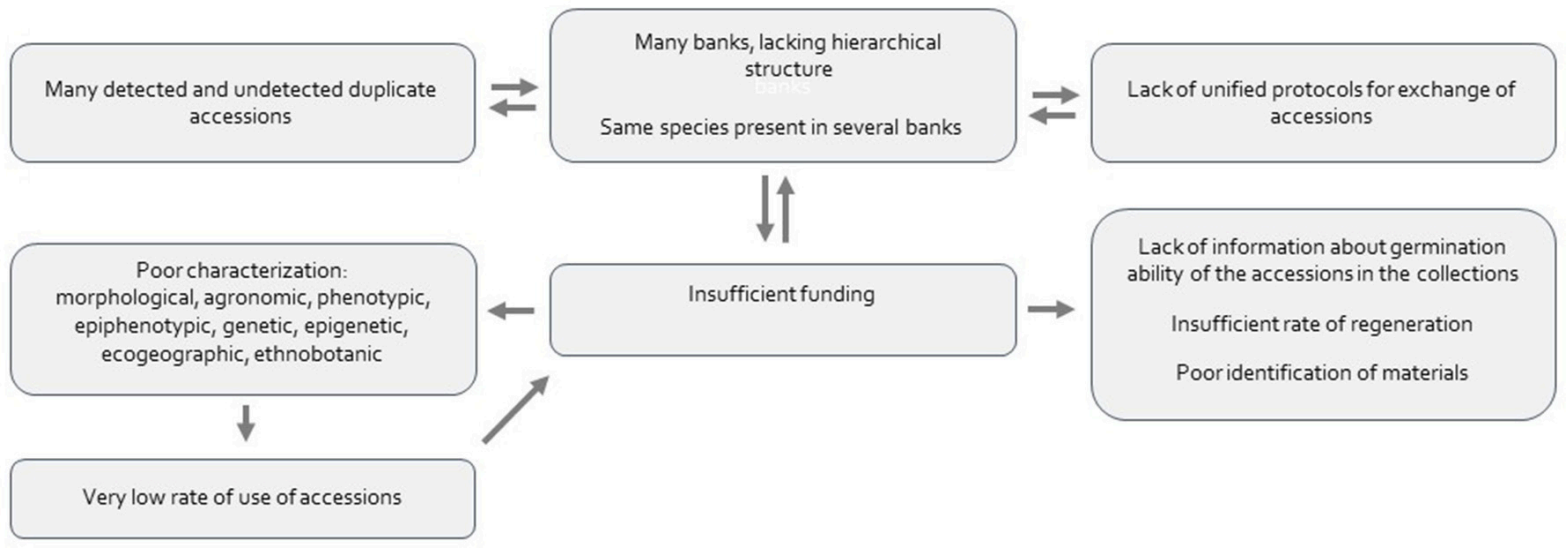
Main shortcomings of the Spanish Plant Genetic Resources Network and relations between them.

**Figure 3:**
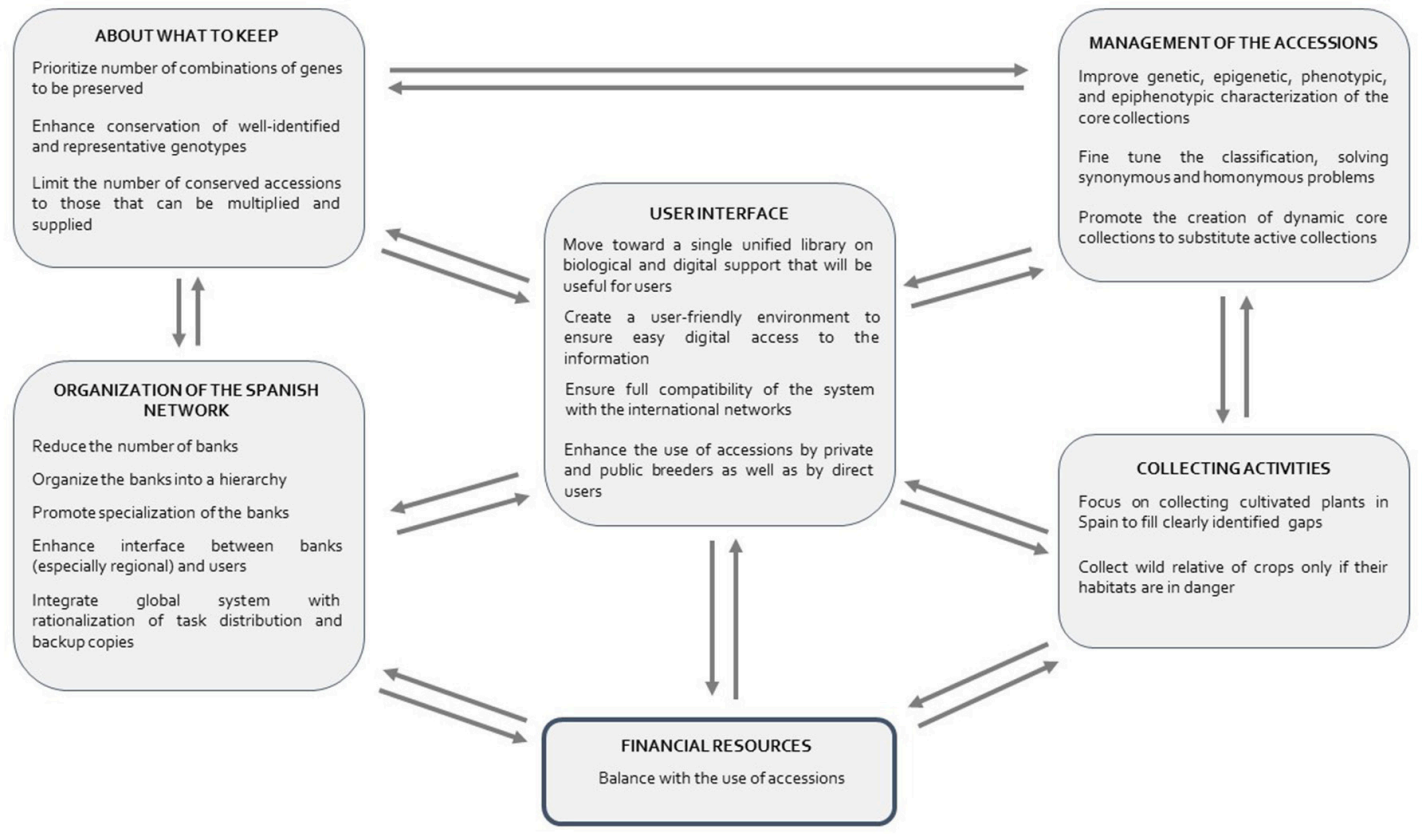
Key points to be improved in the Spanish Plant Genetic Resources Network. As all aspects are related, the corrective actions should be chosen to improve the system with minimum financial increase, favoring the accessions used by breeders and producers. Increase of business and social return of germplasm preserved would help financial returns to the system.

The activities of the Spanish Plant Genetic Resources Network have facilitated the creation of large collections while avoiding the loss of many potential interesting accessions. However, several weaknesses of the system were detected by Guasch et al. (2016) in a workshops series structured by crops, conducted in 2014 and 2015, to perform a Strengths, Weakness, Opportunities and Threats analysis. In our survey of the Network we have confirmed several interconnected weak points, that need substantial improvement (Figure 2), coinciding with the scenario drawn by FAO in other countries (FAO, 2010). In the following sub-sections we describe the weaknesses and interconnections found (Figures 2, 3), and suggest actions for improving the key points.

### Rationalization of the System and Coordination Among Genebanks

#### Weaknesses

As explained above, all the banks were started up by breeders in certain crops and extended to include new species based on the findings in collecting expeditions and on the exchange of accessions with other genebanks. So, many of the same species are kept in different banks. This is mainly because they were set up in the late 1970s and early 1980s, and it was not until 1993 when the former Ministry of Agriculture, Fisheries and Food created the first Conservation and Utilization of Plant Genetic Resources Program and the CRF was assigned the responsibility of documentation. During that period, as mentioned, some of the INIA activities were inefficiently transferred to regional governments, making coordination more difficult. The result was a clear need for profound rationalization and coordination among genebanks.

#### Suggested Actions

The organization of genebanks differs greatly between countries. Sometimes these disparities derive from the differences in biological diversity among countries, but in any case the goal should be to enable the most efficient use of germplasm (Halewood et al., 2018). In Spain, there are some genebanks with similar or repeated activities; the Program for the Conservation and Sustainable Use of Plant Genetic Resources National Network (http://wwwx.inia.es/inventarionacional/Instituciones.asp) comprises 35 institutions, 19 of which maintain seeds. If we were trying to optimize the network, from the scratch, it would be necessary to conceive the needed structure of banks, defining their characteristics, functions, and number. Taking this into account we have to establish the adaptation measures to rearrange the network to fit into the theoretical structure.

The reference bank that houses the backup copies of the Spanish seed collections would still be the CRF. It would be advisable to have active banks specialized in large groups of related crops (cereals, legumes, vegetables, fruit trees, etc.). However, for horticultural crops currently both CITA and COMAV hold a large number of accessions from different sources. Thus, the National Programme should determine the actions to make them work with common specific standards and let them harmonize the activities of the other banks holding also horticultural crops. The main advantage of nested/grouped genebanks, is the ease with which users can access unified data from characterizations trials done under similar conditions. At a third level, there would be other banks, smaller, more focused on the crops grown in specific agro-climatic zones. Familiar with the materials grown in their areas, these banks would have more direct contact with farmers and would know their needs through interacting with them. Thus, farmers working with these genebanks can be trained in the techniques of conservative selection or can provide populations not yet collected. Current banks operating along these lines are the FMA, IMIDA, IMIDRA, MBG-CSIC, etc., which are already playing an important role in the recovery and enhancement of landraces in their respective areas in cooperation with farmers. The following level would be made up of the banks managed by conservation associations. These banks do not perform breeding work; rather, they are dedicated to the collection, conservation, and dissemination of genetic heritage (Jarvis et al., 2011). Standardizing the work done at these banks and linking them to the general network should be a priority. Finally, as public breeders' banks gave rise to the collections, they should be linked to or associated with the network, although maintained with funds obtained in competitive research projects or special calls. We propose the maximum coordination between the different levels to obtain maximum efficiency by avoiding unnecessarily repeated functions.

Part of this work is being done at present as much effort is being put into nesting and organizing genebanks in Spain. To introduce hierarchy into their functioning they are being grouped according to the type of crops they hold (cereals, leguminous, and industrial crops; vegetables; forage and meadow species; and aromatic, medicinal, and wild plants). In each of these broad categories, one genebank coordinates all the genebanks holding these species.

### Conservation: What and How Much Germplasm Do We Need to Conserve?

#### Weaknesses

There are two main problems related with accession conservation: incomplete seed viability information and material regeneration. Not all the genebanks have the capacity to perform regular seed monitoring. For some of the samples of CRF's base collection, initial data on germinability were not available, but the ongoing FAO recommended intermediate tests is allowing to monitor their viability. In some other cases, there are no seed viability data due to the scarce number of seeds conserved. The constant underfunding has led to a bottleneck in regeneration, with the consequent loss of some accessions. These problems are not unique to Spanish collections as they affect many genebanks and all species (FAO, 2010). Thus, it is essential to raise questions such as: Are we able to retain all materials currently stored? or What type of materials must we prioritize: cultivated or wild species, national, or foreign samples?

#### Suggested Actions

We propose to introduce protocols and scoring systems to prioritize *ex situ* conservation of the species cultivated in Spain and their wild relatives, especially if they are endemic or found in threatened habitats. Interest in wild species is growing; multiple examples of their use in breeding (Hajjar and Hodgkin, 2007; McCouch et al., 2013) underline the need to create national *in situ* inventories to encourage conservation of CWR (Maxted et al., 2007). In Spain, an in-depth study on CWR has resulted in a list of species chosen according to their ability to cross with cultivated species, endemicity, and risk of extinction (Rubio et al., 2018). This study can serve as a guide to examine the current state of conservation of these species, and its results should be taken into account for future plans. In addition to landraces and wild species, materials coming from breeding programs (pre-breeding populations, collections of mutant inbreds, improved varieties, obsolete registered varieties, etc.) must also be preserved in gene banks, since they are of special interest for researchers and breeders.

Another uncontested question is if we have to conserve genes or gene combinations. Since landraces evolve to adapt to changing environments, it seems that a rigorous conservation policy should store all genes and combinations of genes that are created over time. This would require adding accessions continually. The other extreme, favored by biotechnology, would be to preserve cloned genes or even only nucleotide sequences that can be reproduced by genetic editing. Since preserving everything is unfeasible, a compromise solution would be to conserve combinations of adaptive genes or those that translate in high yields along with Mendelian genes of interest. The progress of biotechnology will surely guide the balance between these two strategies (Halewood et al., 2018) and will reduce the number of genotypes to be conserved.

How many accessions can we keep in a reasonable way? The conservation of germplasm is inevitably linked to the system's capacity for regenerating the conserved material, and most Spanish genebanks are overwhelmed. To ensure a good representation of the types of germplasm listed above, we must refine our definition of what is really valuable, and we must redesign our organization to be more efficient. Since the real bottleneck lies in regeneration, the distribution of accessions among the different types of genebanks should be optimized. The base collection, with good facilities to conserve accessions at −18°C for long periods of time, should hold the country's complete seed germplasm collection, which must be conserved under good conditions and well-documented. On the other hand, all the active collections (e.g., COMAV and CITA) should focus on part of the base collection. This part would comprise accessions of specific interest and should represent the total diversity of the base collection. These active collections should be dynamic, changing their priorities according to the interest of the different end users, who should suggest the traits for which the variability represented, should be maximized. As new breeding objectives arise, prioritization would change and strategic materials will be regenerated and made available to users. In this way, active collections can be considered a mixture of core collections, each of which has been constructed to meet specific objectives (Byrne et al., 2018; Wambugu et al., 2018). In the end, this is a question of introducing economic criteria in the composition of active collections and rationalizing base collections, which should be broad, without redundancies, and especially well-documented.

### Identification of the Accessions: Delimitation of the Concept of Variety and Classification of Accessions

#### Weaknesses

Efficient organization of a genebank optimizing the search for information requires clear categories for classifying the materials. The concept of accession is not problematic as it refers to a sample taken in a certain area and linked to its respective passport data or to pre-breeding or breeding materials that are kept independent and identified. Following the definition given by Zeven (1998), an autochthonous landrace is a variety with a high capacity to tolerate biotic and abiotic stress, resulting in high yield stability and intermediate yields under a low-input agricultural system. Whereas, there is a consensus to include all materials that farmers have cultivated for a long time in the landrace category, it is often more complicated to ascribe an accession to a particular landrace. The names and definitions of landraces are lax: collectors have generally accepted the information provided by farmers (cultivation time in the area, name, etc.) without checking its reliability, and this has introduced a certain level of uncertainty as synonymous and homonymous occur (Lema et al., 2010). So, it is complex to assign accessions to a variety or a landrace.

#### Suggested Actions

Fortunately, protocols today are much more rigorous (ecogeographic data are included in databases at collection time), but the material stored in genebanks comes from both old and recent sources. Additionally, the primary characterization carried out during multiplication considers only botanical descriptors related to the morphology of the crop and the reproductive cycle. Nevertheless, landraces' main value does not lie solely in primary characterization data; rather it is linked to their role in the ecosystem and to their adaptation to specific territories, cultural practices, and uses. Data about these aspects are much more informative and should be used together with the descriptors of Bioversity International (formerly IBPGR and IPGRI) and/or the International Union for the Protection of New Varieties of Plants (UPOV) to assign an accession to a variety or varietal type. Molecular characterization can also help to determine the degree to which different accessions of, presumably, the same variety are related, as well as to identify alleles of agronomic interest (Prada, 2009).

Accessions must be classified based on exhaustive characterizations by specialists through field trials designed to enable statistical analyses. This is especially important when the names and characteristics of landraces are linked to legal aspects such as conservation varieties or varieties linked to geographical quality labels. The public bodies should look to specialists to define the limits of the varieties; however, we recognize the difficulties in this approach, so we suggest replacing the term variety with varietal type to favor grouping together materials with some key traits in common but variability for many others, as proposed by (Camacho Villa et al., 2005) regarding the definition of crop landrace. In the next future concepts such as “landrace” or “varietal type” need to be reconsidered to ensure that accessions are correctly classified (Casañas et al., 2017).

### Characterization: Thorough Characterization as a Strategy to Encourage Germplasm Use

#### Weaknesses

No characterization data are available for many accessions, greatly hindering the use of the conserved samples. A rough estimation made in 2010 revealed that less than a 5% of the accessions stored in banks have been characterized for quality traits (Romero del Castillo et al., 2010). Also lacking is information about the “epiphenotype” (data about the relationships between the plant and the environment, including ethnobotanical aspects), which in many cases was not recorded at the time of collection.

#### Suggested Actions

Exhaustive phenotyping of the material stored in the genebanks and of new candidates is key, and the relevant data must be publicly accessible in websites as well as through publicly available Application Programming Interfaces (API) that allow for automatic data requests. These conditions will allow researchers to explore easily the phenotypic variability stored in genebanks (Zamir, 2013). The more information available about the materials in a germplasm collection, the more useful the conserved material will be for farmers and/or researchers (McFerson et al., 1996; Engels et al., 2001).

Each accession should be characterized by its morphological traits, which reflect the expression/variation of botanical aspects and are useful for taxonomical classification. However, as mentioned before, it is just as important to provide an accurate description of its agronomic characteristics, its relationship with the environment, other quality-related properties, and its use. This approach would also allow the makeup of the collection to be adapted to users' needs and preferences. At first, farmers and breeders were interested in increasing yields; then we realized that resistance and tolerance to biotic and abiotic stresses were important components of production and adaptation, so genebanks were screened for these aspects (Tanksley and McCouch, 1997). Later, there was also an interest in nutritional quality and the balance of components that would enable the population to be fed with a few species. Now, consumers have incorporated new elements in their decision making, beginning with the product's appearance and continuing on with various additional sensory traits. But multiple other factors are also important (e.g., industrial, cultural, landscape-related, and medicinal factors, as well as the equilibrium of the ecosystems, territorial distribution of the population, etc.). The more information we have about the materials preserved in the base collections, the easier it will be to change the active collections to meet users' demands (core collections in accordance with users' demands, as described above).

Phenotypic and “epiphenotypic” characterization must be complemented with genotypic characterization, especially now that genotyping is already less expensive than good phenotyping. Genotyping allows us to establish the structure of the genetic variation and, in some cases, the geographical migration of an accession (Reif et al., 2005) or to scan a collection to find genes of agronomic interest (Prada, 2009; McCouch et al., 2013; Wambugu et al., 2018), providing elements that can help organize the collections.

To date, ethnobotanical studies have been largely independent from agronomic studies. Moreover, we are only beginning to understand the role of crops in ecosystems because it has been difficult to develop a comprehensive vision of the biosphere. Therefore, genebanks' databases are not usually cross-referenced with information from cultural studies (Meyer, 2015) and are rarely linked with data from studies about ecological, landscape-related, or economic aspects. This is unfortunate because ethnobotanical data can reveal transcendent information about the properties of a variety (Clawson, 1985; Ahmed et al., 2014) without which it would be impossible to fully understand its agricultural and ecological value. Since landraces' future value is related to their cultural aspects and their integration at higher levels, these data should be merged into a single database. In this direction now a project is underway to create a tool that would fill in the gaps between the botanical and ethnobotanical approaches: The Spanish Inventory of Traditional Knowledge related to Agricultural Biodiversity (IECTBA) (Tardío et al., 2018). Furthermore, future collecting missions should include exhaustive questionnaires about the history and culture associated with each of the accessions, as well as its role in the functioning of higher-level ecosystems (Bioversity and The Christensen Fund, 2009).

### Online Information Accessible Through Search Engines: Seed Banks as Libraries Storing Information in Biological and Digital Formats

#### Weaknesses

Without adequate public databases, information about materials held in genebanks cannot be accessed. In Spain the most complete database is the National Inventory that holds data of a big proportion of the accessions conserved in Spanish genebanks. However, most part of this information is about passport data. With regard to characterization data, in most cases the existing information is not available to community of users in easily accessible formats, as there are no databases that exhaustively collect characterization or ecogeographic data. Furthermore, the lack of standardization of descriptors between datasets greatly complicates the creation of shared databases.

#### Suggested Actions

Considerable efforts have already been devoted at an international level to merging documentation from different sources. The most complete database worldwide, Genesys (https://www.genesys-pgr.org/en/welcome) (Arnaud et al., 2010), contains passport data for more than 3.5 million accessions from 458 institutions. This first step enables wide-reaching searches for information on plant genetic resources.

In Spain, the CRF manages the documentation system for Spanish germplasm published in the National Inventory. At present, the INIA is funding a project to create an informatics platform to optimize documentation with the aim of facilitating the management of information within the CRF and adding characterization data and images to supplement the passport data. This informatics platform will also allow duplicated accessions to be easily identified and the information about the germplasm preserved in all Spanish institutions to be easily accessed.

This system would benefit from including all data from publicly funded research and development projects on food and agriculture to expand the characterization of germplasm currently available and thus favor its use. As the researchers who receive the material from the genebanks are those who collect these data, arranging for their findings to remain confidential for a short period would allow them to take advantage of this information before transferring it to the public domain.

In addition, in some cases genebanks' participation in research projects has allowed them to get access to the genotype of some of their accessions. Analyzing these data can benefit the management of these accessions, for example by helping to identify duplicates, to structure collections, or to create core collections. Although it would be feasible to include these data in the genebanks' databases, the benefits for the genebanks themselves are unclear because their staffs are generally not trained in interpreting raw data from genotyping.

Beyond eliminating barriers to create a single large database, it is important to accelerate the addition of data from genotypic, epigenetic, phenotypic, and epiphenotypic characterization to the passport data. Additionally, the use of digital object identifier (DOI) system would connect different data sources and increase traceability of accession use. Merging all these data is a medium-term objective that will require time and efforts, and we should also strive to ensure that new accessions have these levels of information. The new informatics platforms must facilitate the storage and management of these four levels of information. Also, these platforms must have a user-friendly interface not only for researchers or breeders but also for farmers, farmers' associations, etc. Map search engines dealing with ecogeographic and epiphenotypic layers should also be developed.

### Funding of Germplasm Banks

#### Weaknesses

Genebanks belonging to the National Network are primarily funded through competitive research grants from the INIA and their own institutions. The first result of the above-mentioned RD199/2017 was the approval of its Action Plan for Conservation and Sustainable Use of Plant Genetic Resources for Food and Agriculture (2018–2022), which will be the starting point for new calls based on a newly organized, crop-driven collection network, connected with the working groups of the European Cooperative Program for Plant Genetic Resources (ECPGR). This funding must cover the “permanent activities,” which include ensuring the sustainable conservation of plant genetic resources, promoting their regeneration and primary characterization, and delivering materials to users, based on the accomplishment of specific standards. In addition, the institutions in which the banks are located contribute by providing facilities and equipment (conservation chambers, phytotrons, greenhouses, experimental fields, etc.) as well as permanent staff in some cases. This arrangement has resulted in a steady state that allows for the maintenance of the collections, but is insufficient for adequate regeneration and phenotyping and provides no funds whatsoever for genotyping.

#### Suggested Actions

We must find alternatives that provide genebanks with additional funding. One such alternative is to charge a fee for each accession delivered. Several genebanks already use this measure, which helps to filter out unjustified requests for germplasm. Its usefulness, however, depends on the bureaucratic requirements for its application and the fees charged. In Europe, the Leibniz Institute of Plant Genetics and Crop Plant Research (IPK) decided in July 2016 to introduce a handling fee for distributing its samples. Similarly, the World Vegetable Centre (AVRDC) also introduced different rates depending on the country and requesting entity and the type of material (landrace/breeding material) to contribute to the expenses of maintaining the collection, the fifth largest in the world in the number of accessions preserved. An exhaustive economic study by the AVRDC quantifies the large expenses involved in maintaining germplasm (Schreinemachers et al., 2014). Despite a reluctance to apply fees in some areas and the inconvenience involved, we think there are sufficient arguments for using this approach in our genebanks.

Another alternative is co-financing, as is done with other public services. Alternative financing options could be explored with public and private breeding companies. Expanded characterization data saves work for breeders and could command higher prices. A further option would be for users to pay according to the benefit obtained, which is feasible because the material can be tracked by studying the genomes. Finally, contributions to the conservation of seeds could be sought from the general public through sponsorship, etc. In an analysis of vulnerability of plant genetic resources conserved *ex situ*, Fu (2017) suggests alternatives such as partnership with the private sector. Another indirect way to “socialize” the materials in genebanks is through the creation of public, private, or mixed companies that produce and distribute “heirloom” or “vintage” seeds or seedlings to be used in family gardens. This is already a flourishing business in some countries.

In Spain, there is a lack of correspondence between the conservation of cultivated germplasm, which is mainly managed by public organisms, and plant breeding, which is mainly managed by private companies. Entities responsible for conserving germplasm are chronically underfunded and can barely get by through public subsidies, while breeding generates significant profits. The access under the multilateral system provided by a standard material transfer agreement includes a contribution to the profit-sharing fund, but its functioning is under discussion and the funds are not directed to the genebanks providing the seeds. It is difficult to impose solutions, but we must seek approaches that allow us to move toward a greater presence of genebanks in the business sector.

## The Usefulness of Genebanks

Genebanks were created to ensure the survival of combinations of genes (or individual genes) that were present when we realized that the biodiversity of crops had stopped increasing and started decreasing (Fu, 2017). Despite globalization, our planet has a variety of habitats and in each habitat, the maximum productive efficiency for each species is achieved with specific combinations of genes. This means that low-input agriculture faces the challenge of finding the most adapted genotypes for each environment. As we tend to decrease the amounts of inputs aimed at equalizing environments (lower irrigation and fertilization, less herbicides and pesticides, etc.), we need genotypes that are better adapted to particular environments. Many of these genotypes or their precursors are undoubtedly preserved in genebanks.

The situation is similar at the global level. Humans' influence has not only led to changes at the level of particular habitats; rather, it is also bringing about great changes in the global habitat. Warming is changing the climatic and even geographical characteristics of many areas (Olesen and Bindi, 2002). Breeders specialized in obtaining new varieties to be cultivated in large areas of the world must also update the combinations of genes that were so successful during the twentieth century, to adapt to new conditions (Ceccarelli and Grando, 1996; Chapman et al., 2012). What has been said for “micro” adaptations also serves for “macro” adaptations. Again, gene banks hold the resources for addressing this goal (Lema et al., 2010; Wambugu et al., 2018), but these resources are useful only if they are well-documented and easily accessible.

The justification for our investments in genebanks must be based on the use of the materials they contain to meet new demands such as increased productivity, resistance, and adaptation to low input conditions, as well as improved sensory and nutritional characteristics, industrial, medicinal, landscape, and social values, etc. The genebanks also have a role in educating new generations about the importance of preserving biodiversity and the best way to go about this mission. In any case, although the formats evolve, genebanks will continue to be information libraries where to look for the meaning of genes. The greater the quantity and quality of the information stored and the better the engines we have to process and filter it, the greater the utility of the bank. In turn, the degree of utility, in the medium or long term, will determine their survival (Byrne et al., 2018).

## The Future of the Genebanks

Advances in biotechnology techniques pave the way to new scenarios that reach beyond the reflections in this article into the future. The massive sequencing of many accessions of many crops has generated huge quantities of data. One example is the work done in rice (*Oryza sativa* L.) at the International Rice Research Institute, in which numerous researchers have collaborated (McCouch et al., 2012). The availability of this information has in turn enabled breeding materials to be developed: mapping populations, homozygous materials essential for resequencing and allele identification, etc., in short, materials other than heterogeneous populations normally kept in banks. Before these advances, the focus was on landraces, wild relatives, passport data, and primary characterization; now the scenario is much more complex. Users, especially researchers and breeders, need not only accessions but also as much associated information as possible. In addition, we are beginning to glimpse the possibilities of gene editing, so the importance of physically preserving biological information (seeds or propagules) may be declining.

Genebanks must start re-thinking their mission, especially considering users' needs and presenting information in an accessible and useful way (Van Treuren and van Hintum, 2014). To advance in this direction, genebanks in Spain and around the world should assume their changing role and integrate into worldwide initiatives that have been underway for years, including such measures as **s**trength collaborations between genebanks and the community of users. Users can contribute in many ways to the sustainability of the banks, for example, by regenerating accessions, supporting collecting missions, developing pre-breeding materials that can be shared by the scientific community, contributing their phenotyping and genotyping data to improve the information in databases and specific information portals, etc. The bidirectional flow of information must be encouraged, moving beyond the present arrangement in which information flows only from genebanks to users. In short, users must have a role in the maintenance of germplasm collections (Wambugu et al., 2018).

Genebanks must present the information in a way that meets the specific needs and interests of the users, beyond the passport data. Thus, genebanks should develop portals with specific information for their users, for example, for lettuce (*Lactuca sativa* L.) breeders interested in resistance, researchers working with landraces of a particular crop, etc. The information provided should be organized differently in each case to ensure the maximum usefulness for the user's purpose. The CGN of The Netherlands has developed prototypes of these portals for lettuce (pgrportal.nl/lettuce) and potato (pgrportal.nl /potato).

Genebanks should incorporate pre-breeding materials developed by researchers in their collections, because landraces are not the only useful source of germplasm, although including different types of collections may require different conservation and distribution protocols. Besides, we should consider the possibility of the participation of the researchers themselves and seed companies interested in the maintenance of these materials. Likewise, these materials can be transferred through specific transfer agreements.

The large quantity of data about origins and characterization that is available in genebanks should be interconnected with efficiently genotyping data, annotation of genomes, etc., from the scientific community. The question “How will genebanks use and provide access to genomic data and how will genomic information resources give access to genebank data and materials?” has already been discussed recently (Finkers et al., 2015). Bioinformatics technologies allow databases to be interconnected through the use of publicly defined Application Programming Interfaces and DOIs. The automated interconnection of different data sources that deal with different aspects of the collections (e.g., passport or genomic data) should add value to the biological materials stored. For example, when the name of a specific gene is entered, the application can provide a list of genebank accessions containing different alleles as well as the available phenotyping data and passport data. This information can be invaluable for users requesting accessions of interest. McCouch et al. (2013) suggested building databases that integrate passport information, genotyping data, and phenotyping data, and coordinated work on this initiative is underway.

## Genebanks in the Era of Genetic Editing

With the aim of going further and trying to elucidate the role of genebanks in the era of genetic editing, we consulted some significant Spanish biotechnologists and geneticists. The main message we got from them is that genebanks store a high number of genes and gene combinations created by evolution and that, at present, cannot be produced by biotechnology or genetic editing techniques. Although this would be perhaps the case in a future, variability reservoirs will continue to be indispensable for determining the gene function.

Nevertheless, genetic editing techniques and other current biotechnology tools will allow us to take advantage of the information held in genebanks in a more efficient and fast way, contributing to the rationalization and running. Genotyping of the collections, both at low and high density, will allow the identification of duplicates, the establishment of varietal limits, the estimation of the population variability, the identification of haplotypic blocks, etc., that is, to rationalize the genebanks in a scenario where genotyping is much cheaper than phenotyping. Taking advantage of sequencing progress, genebanks should be collections of allelic variants of genes responsible of characteristics of interest and should provide information about the sequence associated to phenotype.

Future genebanks should also conserve products of genetic editing and transgenics, as well as the associated information. Finally, they should conserve DNA, as well as all the information related to the sequences of the corresponding genomes. It seems then clear than genebanks, mainly historically born from breeder's collections and then segregated into individual new bodies, should now associate to research and breeding institutions to get all their sense.

## Final Considerations

Spanish genebanks cannot apply all the above mentioned correcting measures in the short term, but we should start looking in this direction and the most basic aspects discussed should be prioritized. In fact, some genebanks at an international level are already genotyping a large number of accessions in their collections by participating in research projects. In the ongoing project H2020 G2P-SOL (www.g2p-sol.eu), more than 45,000 accessions of major solanaceous crops [tomato, pepper (*Capsicum annuum* L.), eggplant (*Solanum melongena* L.), and potato (*Solanum tuberosum* L.)] from 12 countries in Europe, Asia, and America are being genotyped. Using these genotyping and passport data, core collections are being built. The joint study of all these data will allow to elucidate the genetic relationships between all these inputs (practically all the collections of these crops worldwide), identify alleles and shared regions, locate the places where to look for new variation, determine the value of the different collections, identify accessions with rare alleles of interest, associate genotypes with phenotypes, etc. These actions represent an advance in applying the guidelines mentioned along the manuscript and we hope that models like that will inspire the Spanish Plant Genetic Resources Network to adapt to changing technologies and demands. The recently constituted Commission of the National Program for the Conservation and Utilization of Plant Genetic Resources for Agriculture and Food, besides structuring the Network, updating information on Spanish genebanks, and streamlining access to their repositories, should address the international challenge of rethinking them.

## Author Contributions

MD and FC conceived the project, organized a specific meeting for discussion on this matter, and supervised the manuscript. LD, IM, MC, CM, JC, JS, AR, GA, JP, SS, JB, and JV attended the meeting specifically focused on this matter and critically reviewed the manuscript. LG critically reviewed the manuscript.

### Conflict of Interest Statement

The authors declare that the research was conducted in the absence of any commercial or financial relationships that could be construed as a potential conflict of interest.

## References

[B1] Ahmed S. Stepp J. R. Orians C. Griffin T. Matyas C. Robbat A.. (2014). Effects of extreme climate events on tea (*Camellia sinensis*) functional quality validate indigenous farmer knowledge and sensory preferences in tropical China. PLoS ONE 9:e109126. 10.1371/journal.pone.0109126 25286362 PMC4186830

[B2] Arnaud E. Dias S. Mackay M. Cyr P. Gardner C. Bretting P. (2010). A global portal enabling worldwide access to information on conservation and use of biodiversity for food and agriculture, in Information and Communication Technologies for Biodiversity Conservation and Agriculture, eds Maurer Lisa L. Tochtermann K. (Aix-la-Chapelle: Shaker Verlag), 168–180.

[B3] Bioversity and The Christensen Fund (2009). Descriptors for Farmers' Knowledge of Plants. Bioversity International, Rome, Italy and The Christensen Fund, Palo Alto

[B4] Bueno M. A. Alaman M. C. (1982). Los bancos de germoplasma: una estrategia para el futuro. Rev. Ext. Agrar. 21, 65–71.

[B5] Byrne P. F. Volk G. M. Gardner C. Gore M. A. Simon P. W. Smith S. (2018). Sustaining the future of plant breeding: the critical role of the USDA-ARS national plant germplasm system. Crop Sci. 58, 451–468. 10.2135/cropsci2017.05.0303

[B6] Camacho Villa T. C. Maxted N. Scholten M. Ford-Lloyd B. (2005). Defining and identifying crop landraces. Plant Genet. Resour-C. 3, 373–384. 10.1079/PGR200591

[B7] Cartea M. E. Picoaga A. Soengas P. Ordás A. (2003). Morphological characterization of kale populations from northwestern Spain. Euphytica 129, 25–32. 10.1023/A:1021576005211

[B8] Casals J. Casañas F. Simó J. (2017). Is it necessary to continue to collect genetic crop resources in the Mediterranean area? A case study in Catalonia. Econ. Bot. 71, 330–341. 10.1007/s12231-017-9392-0

[B9] Casañas F. Bosch L. Sánchez E. Romero del Castillo R. Valero J. Baldi M. (1997). Collecting, conservation and variability of Ganxet common bean (*Phaseolus vulgaris* L.). Plant Genet. Resour. 112, 105–106.

[B10] Casañas F. Simó J. Casals J. Prohens J. (2017). Toward an evolved concept of landrace. Front. Plant Sci. 8:145. 10.3389/fpls.2017.00145 28228769 PMC5296298

[B11] Cebolla-Cornejo J. Roselló S. Nuez F. (2013). Phenotypic and genetic diversity of Spanish tomato landraces. Sci. Hortic. 162, 150–164. 10.1016/j.scienta.2013.07.044

[B12] Ceccarelli S. Grando S. (1996). Drought as a challenge for the plant breeder. Plant Growth Regul. 20, 149–155. 10.1007/BF00024011

[B13] Chapman S. C. Chakraborty S. Dreccer M. F. Howden S. M. (2012). Plant adaptation to climate change—opportunities and priorities in breeding. Crop Pasture Sci. 63, 251–268. 10.1071/CP11303

[B14] Clawson D. L. (1985). Harvest security and intraspecific diversity in traditional tropical agriculture. Econ. Bot. 39, 56–67. 10.1007/BF02861175

[B15] De-Zhu L. Pritchard H. W. (2009). The science and economics of *ex situ* plant conservation. Trends Plant Sci. 14, 614–621. 10.1016/j.tplants.2009.09.005 19818672

[B16] Engels J. M. M. Rao V. R. Brown A. H. D. Jackson M. (2001). Managing Plant Genetic Diversity. New York, NY: Cabi.

[B17] Engels J. M. M. Thormann I. (2007). Horticultural genetic resources collections: Their characteristics, strengths and weaknesses. Acta Hortic. 760, 33–42. 10.17660/ActaHortic.2007.760.2

[B18] FAO (2010). The Second Report on the State of the World's Plant Genetic Resources for Food and Agriculture. Rome: FAO Available online at: http://www.fao.org/docrep/013/i1500e/i1500e.pdf

[B19] Finkers R. Chibon P. Y. van Treuren R. Visser R. van Hintum T. (2015). Genebanks and genomics: how to interconnect data from both communities? Plant Genet. Resour-C. 13, 90–93. 10.1017/S1479262114000689

[B20] Fowler C. Hodgkin T. (2004). Plant genetic resources for food and agriculture: assessing global availability. Annu. Rev. Env. Resour. 29, 143–179. 10.1146/annurev.energy.29.062403.102203

[B21] Fu Y. B. (2017). The vulnerability of plant genetic resources conserved *ex situ*. Crop Sci. 57, 2314–2328. 10.2135/cropsci2017.01.0014

[B22] Gepts P. (2006). Plant genetic resources conservation and utilization. Crop Sci. 46, 2278–2292. 10.2135/cropsci2006.03.0169gas

[B23] Gómez O. J. Blair M. W. Frankow-Lindberg B. E. Gullberg U. (2005). Comparative study of common bean (*Phaseolus vulgaris* L.) landraces conserved *ex situ* in genebanks and *in situ* by farmers. Genet. Resour. Crop Evol. 52, 371–380. 10.1007/s10722-005-2249-x

[B24] Guasch L. M. Fajardo J. de la Rosa L. (2016). Informe de la reunión de coordinación de investigación agraria INIA-Comunidades Autónomas: propuesta de actuación sobre las colecciones de recursos fitogenéticos. Reunión interregional de I+D en agricultura. Logroño febrero 2016, 1–29.

[B25] Hajjar R. Hodgkin T. (2007). The use of wild relatives in crop improvement: a survey of developments over the last 20 years. Euphytica 156, 1–13. 10.1007/s10681-007-9363-0

[B26] Halewood M. Chiurugwi T. Sackville Hamilton R. Kurtz B. Marden E. Welch E.. (2018). Plant genetic resources for food and agriculture: opportunities and challenges emerging from the science and information technology revolution. New Phytol. 217, 1407–1419. 10.1111/nph.14993 29359808

[B27] Hancock J. F. (2004). Plant Evolution and the Origin of Crop Species. Cambridge: CABI Publishing.

[B28] Jarvis D. I. Hodgkin T. Sthapit B. R. Fadda C. Lopez-Noriega I. (2011). An heuristic framework for identifying multiple ways of supporting the conservation and use of traditional crop varieties within the agricultural production system. Crit. Rev. Plant Sci. 30, 125–176. 10.1080/07352689.2011.554358

[B29] Khoury C. K. Achicanoy H. A. Bjorkman A. D. Navarro-Racines C. Guarino L. Flores-Palacios X. (2016). Origins of food crops connect countries worldwide. P. R. Soc. B-Biol. Sci. 283:2060792 10.1098/rspb.2016.0792

[B30] Khoury C. K. Bjorkmann A. D. Dempewolf H. Ramirez-Villegas J. Guarino L. Jarvis A.. (2014). Increasing homogeneity in global food supplies and the implications for food security. Proc. Natl. Acad. Sci. U.S.A. 111, 4001–4006. 10.1073/pnas.1313490111 24591623 PMC3964121

[B31] Lema M. P. Soengas P. Velasco M. Cartea M. E. (2010). Las razas locales como reservorio de variabilidad genética, in Mejora genética y recursos fitogenéticos: Nuevos Avances en La Conservación y Utilización de los Recursos Fitogenéticos, eds Carrillo J. M. Díez M. J. Pérez de la Vega M. Nuez F. (Madrid: Ministerio de Medio Ambiente y Medio Rural y Marino), 547–580.

[B32] Maxted N. Scholten M. Codd R. Ford-Lloyd B. (2007). Creation and use of a national inventory of crop wild relatives. Biol. Conserv. 140, 142–159. 10.1016/j.biocon.2007.08.006

[B33] McCouch S. Baute G. J. Bradeen J. Bramel P. Bretting P. K. Buckler E. (2013). Feeding the future. Nature 499, 23–24. 10.1038/499023a 23823779

[B34] McCouch S. McNally K. L. Wang W. Sackville Hamilton R. (2012). Genomics of gene banks: a case study in rice. Am. J. Bot. 99, 407–423. 10.3732/ajb.1100385 22314574

[B35] McFerson J. R. Lamboy W. F. Kresovich S. (1996). Assessing user perceptions of genetic resource collections in crucifer crops. Crop Sci. 36, 831–838. 10.2135/cropsci1996.0011183X003600040001x

[B36] Meyer R. S. (2015). Encouraging metadata curation in the diversity seek initiative. Nat. Plants 1:15099. 10.1038/nplants.2015.99 27250263

[B37] Negri V. Tiranti B. (2010). Effectiveness of in situ and ex situ conservation of crop diversity. What a *Phaseolus vugaris* L. landrace case study can tell us. Genètica 138, 985–998. 10.1007/s10709-010-9485-5 20835753

[B38] Olesen J. E. Bindi M. (2002). Consequences of climate change for European agricultural productivity, land use and policy. Eur. J. Agron. 16, 239–262. 10.1016/S1161-0301(02)00004-7

[B39] Prada D. (2009). Molecular population genetics and agronomic alleles in seed banks: searching for a needle in a haystack? J. Exp. Bot. 60, 2541–2552. 10.1093/jxb/erp130 19451185

[B40] Reif J. C. Melchinger A. E. Frisch M. (2005). Genetical and mathematical properties of similarity and dissimilarity coefficients applied in plant breeding and seed bank management. Crop Sci. 45, 1–7. 10.2135/cropsci2005.0001

[B41] Romero del Castillo R. Sabaté J. Plans M. Casañas F. (2010). Evaluación para características de calidad, in Mejora Genética y Recursos Fitogenéticos: Nuevos Avances en la Conservación y Utilización de los Recursos Fitogenéticos, eds Carrillo J. M. Díez M. J. Pérez de la Vega M. Nuez F. (Madrid: Ministerio de Medio Ambiente, Medio Rural y Marino), 383–420.

[B42] Rubio M. L. Torres E. Parra-Quijano M. de la Rosa L. Fajardo J. Iriondo J. M. (2018). National inventory and prioritization of crop wild relatives in Spain. Genet. Resour. Crop Evol. 65, 1237–1253. 10.1007/s10722-018-0610-0

[B43] Santalla M. Rodiño A. de Ron A. M. (2002). Allozyme evidence supporting southwestern Europe as a secondary center of genetic diversity for common bean. Theor. Appl. Genet. 104, 934–994. 10.1007/s00122-001-0844-6 12582598

[B44] Schreinemachers P. Ebert A. W. Wu M. H. (2014). Costing the *ex situ* conservation of plant genetic resources at AVRDC - The World Vegetable Center. Genet. Resour. Crop Evol. 61, 757–773. 10.1007/s10722-013-0070-5

[B45] Soengas P. Cartea M. E. Velasco P. Padilla G. Ordás A. (2008). Morphologic and agronomic diversity of *Brassica napus* crops. J. Amer. Soc. Hort. Sci. 133, 48–54.

[B46] Tanksley S. D. McCouch S. R. (1997). Seed banks and molecular maps: unlocking genetic potential from the wild. Science 277, 1063–1066. 10.1126/science.277.5329.1063 9262467

[B47] Tardío J. Pardo de Santayana M. Morales R. Molina M. Aceituno L. (2018). Inventario Español de Conocimientos Tradicionales Relativos a la Biodiversidad Agrícola Volumen I. Madrid: Ministerio de Agricultura, Pesca y Alimentación.

[B48] Van de Wouw M. Kik C. van Hintum T. van Treuren R. Visser B. (2009). Genetic erosion in crops: concept, research results and challenges. Plant Genet. Resour. 8, 1–15. 10.1017/S1479262109990062

[B49] Van Treuren R. van Hintum T. (2014). Next-generation genebanking: plant genetic resources management and utilization in the sequencing era. Plant Genet. Resour. 12, 298–307. 10.1017/S1479262114000082

[B50] Veteläinen M. Negri V. Maxted N. (2009). European Landraces: On-Farm Conservation, Management and Use. Bioversity Technical Bulletin N15. Rome: Bioversity International Available online at: https://www.bioversityinternational.org/fileadmin/_migrated/uploads/tx_news/European_landraces__on-farm_conservation__management_and_use_1347.pdf

[B51] Wambugu P. W. Ndjiondjop M. N. Henry R. J. (2018). Role of genomics in promoting the utilization of plant genetic resources in genebanks. Brief. Funct. Genomics 17, 198–206. 10.1093/bfgp/ely014 29688255 PMC5967547

[B52] Zamir D. (2013). Where have all the crop phenotypes gone? PLoS Biol. 11:e1001595. 10.1371/journal.pbio.1001595 23824246 PMC3692434

[B53] Zeven A. C. (1998). Landraces: a review of definitions and classifications. Euphytica 104, 127–139. 10.1023/A:1018683119237

